# The Shock of Shatter: Understanding Silique and Silicle Dehiscence for Improving Oilseed Crops in Brassicaceae

**DOI:** 10.1002/pld3.70058

**Published:** 2025-04-13

**Authors:** Justin B. Nichol, Shakshi A. Dutt, Marcus A. Samuel

**Affiliations:** ^1^ Department of Biological Sciences University of Calgary Calgary Alberta Canada

**Keywords:** agriculture production, cell wall, lignification, oilseed crops, silique dehiscence, yield increases

## Abstract

Silique dehiscence, despite being an essential physiological process for seed dispersal for dehiscent fruits, is disadvantageous for the agricultural industry. While crops have been selected against the expression of natural, spontaneous shattering to protect the seeds for harvest, fruit dehiscence in the field can be promoted through abiotic factors such as wind, drought, and hail that can be detrimental in reducing crop yield and profitability. In crops like canola, pennycress, and *Camelina*, this impact could be as high as 50%, creating economic losses for both the industry and the economy. Mitigating the effects of fruit dehiscence is crucial to prevent seed loss, economic loss, and the persistence of volunteer plants, which interfere with crop rotation and require increased weed control. Developing agronomic traits through genetic manipulation to enhance the strength of the fruiting body can prevent seed dispersal mechanisms from occurring and boost yield efficiency and preservation. Current research into this area has created mutant plants with indehiscent fruits by reducing allele function that determines the identity of the various anatomical layers of the fruit. Future genetic approaches may focus on strengthening siliques by enhancing secondary cell walls through either increased lignification or reducing cell wall‐degrading enzymes to achieve shatter tolerance. This review focuses on improving our knowledge within members of the Brassicaceae family to create a better understanding of silique/silicle dehiscence for researchers to establish a groundwork for broader applications across diverse crops. This knowledge will directly lead to improved agricultural productivity and ensure a stable food supply, addressing global challenges the world is facing.

## 1 Introduction

Silique dehiscence is necessary for seed dispersal at the end of a dehiscent plant's lifecycle. While this process is critical for seed dispersal, this trait is disadvantageous in cropping systems due to the need to retain the seeds in the siliques for harvesting. Therefore, cultivated crops have been naturally selected or bred against the spontaneous seed dispersal trait. Despite this, premature dispersal can still occur because of abiotic factors, such as wind, drought, and hail that can be detrimental to crop yield. Mitigating the effects of fruit dehiscence is important because seed loss, in addition to economic losses, can lead to volunteer seeds persisting in the field, giving rise to plants that interfere with crop rotation and herbicide selection (Price et al. 1996). These volunteer plants have been shown to demand increased weed control measures and even have phytotoxic effects on the subsequent generations (Price et al. 1996; Gan et al. 2008; The Canola Council of Canada 2024). Preventing silique and silicle dehiscence by developing novel agronomic traits in siliques can have several benefits, ranging from increased yield efficiencies, yield preservation, and reduction of volunteer plants (Price et al. 1996; Gan et al. 2008; The Canola Council of Canada 2024). Within the Brassicaceae system (Zuñiga‐Mayo et al. 2018), the model plant *Arabidopsis thaliana* has been used primarily to translate the regulatory network governing silique development to canola and other cash crops (Liljegren et al. 2000; Rajani and Sundaresan 2001; Ogawa et al. 2009; Groszmann et al. 2011; Lenser and Theißen 2013; Braatz et al. 2018; Zuñiga‐Mayo et al. 2018; Di Vittori et al. 2019). One aspect of this dehiscence trait that has not been explored sufficiently is the lignification of specific valve layers that are critical for dehiscence. The fundamental research in *A. thaliana* can therefore be used as a foundation for the elucidation of genes associated with lignification in addition to other processes related to fruit dehiscence. This is of utmost importance due to our ever‐changing climate and exponential population growth, making it crucial to increase food security and sustainability.

## 2 Silique and Silicle Morphology in Brassicaceae

Much of our understanding of silique dehiscence is based on previous work done in *A. thaliana*, because it is a closely related species to many important oil and cash crops in the Brassicaceae family. Therefore, it is an ideal candidate for understanding this complex fruit development–associated process. Researchers have classified the development of the flowering and fruiting body into 20 different stages, with flower development beginning at Stages 1–12 and silique development from Stages 13–20 (Smyth et al. 1990). The developmental stages are based on landmark morphological events that define each stage (Smyth et al. 1990). An in‐depth look into fruit development within *Arabidopsis* and of the aforementioned stages has been conducted by Roeder and Yanofsky (2006) and Herrera‐Ubaldo and Folter (2022). Other work focuses primarily on silique development as days post anthesis (DPA), which refers to flower bud opening, and also days after pollination (DAP), after the flower has been pollinated (Vivian‐Smith and Koltunow 1999; Mizzotti et al. 2018; Nichol and Samuel 2024). Fundamentally, these two classification systems offer similar results because DPA and DAP occur concomitantly in *A. thaliana* (Herrera‐Ubaldo and Folter 2022). The silique matures from a gynoecium, containing the stigma, style, ovary, and gynophore (Ferrándiz et al. 1999). In brief, the gynoecium of *A. thaliana* is syncarpous with an axile ovary placentation, which contains two locules (Herrera‐Ubaldo and Folter 2022). For a more in‐depth description of gynoecium development, refer to the review by Herrera‐Ubaldo and Folter (2022), which offers a complete overview of the process.

**FIGURE 1:**
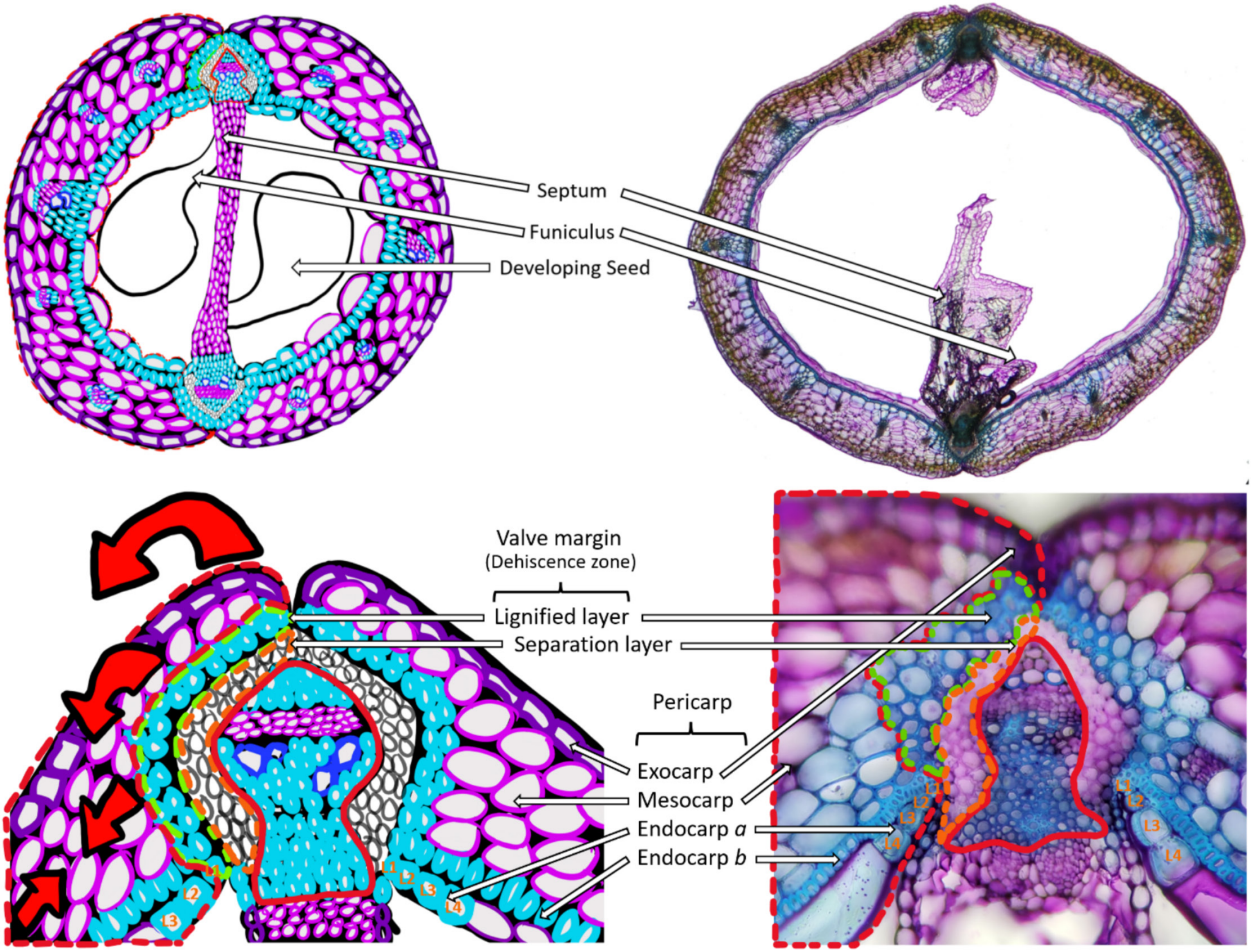
An illustrative figure of a silique cross‐section and *B. napus* cross‐section. The two images are representative of a silique cross‐section. The illustrative images are false‐colored to mimic toluidine blue O staining, with the pink/purple color representing the primary cell wall and the blue‐colored cells indicating secondary cell wall lignification. The real *B. napus* cross‐sections are stained with toluidine blue O. The red dotted line indicates the valve. The green dotted line indicates the lignified layer. The orange dotted line indicates the separation layer. The red line indicates the replum.

In *Arabidopsis*, *Cardamine hirsuta*, and the *Brassicas*, the ovary space of the silique contains two chambers separated by a false partition, the septum, which attaches between two replum regions on either side (Spence et al. 1996; Dinneny et al. 2005) (Figure 1). The walls of the ovary space are made up of two crescent‐shaped valves that merge at the replum's central seam via the valve margins that run in parallel up the length of the silique from the gynophore to the base of the style (Spence et al. 1996; Dinneny et al. 2005) (Figure 1). Seeds develop within the two chambers of the ovary space, protected by the valve, and fed through the funiculus that develops from the septum (Spence et al. 1996; Dinneny et al. 2005) (Figure 1). The replum provides the main vasculature for the developing silique (Alvarez and Smyth 2002). The valve and replum merge at the valve margin, which consists of two distinct cell layers, the separation layer (SL) and the lignified layer (LL) (Ferrándiz et al. 1999; Liljegren et al. 2004; Roeder and Yanofsky 2006) (Figure 1). The SL is formed by two layers of cells that run along the length of the replum and is the site where specific cell wall degrading enzymes act (Rajani and Sundaresan 2001; Liljegren et al. 2004; Roeder and Yanofsky 2006) (Figure 1). This is often referred to as the dehiscence zone (DZ), where separation occurs allowing the silique to open (Spence et al. 1996) (Figure 1). The LL undergoes lignification on the outer side of the SL (Liljegren et al. 2004; Roeder and Yanofsky 2006) (Figure 1).

The valve pericarp consists of three different cell layers that vary in size, consisting of the exocarp, mesocarp, and endocarp (Rajani and Sundaresan 2001; Liljegren et al. 2004; Roeder and Yanofsky 2006) (Figure 1). The exocarp is the outermost cell layer that consists of a file of rectangular‐shaped cells, which contain a thick layer of epicuticular wax and a waxy cuticular layer protecting the silique and seeds from biotic and abiotic factors, such as pathogens, pests, and intense weather conditions (Rajani and Sundaresan 2001; Liljegren et al. 2004; Roeder and Yanofsky 2006) (Figure 1). The exocarp also contains stomata required for transpiration and respiration (Rajani and Sundaresan 2001; Liljegren et al. 2004; Roeder and Yanofsky 2006). The mesocarp is the middle cell layer that comprises most of the valve tissue, usually consisting of three layers of thin‐walled parenchyma cells (Rajani and Sundaresan 2001; Liljegren et al. 2004; Roeder and Yanofsky 2006) (Figure 1). It is the primary photosynthetic tissue layer within the valve and serves as an important cell layer during silique drying and dehiscence, providing the tensile forces necessary on the SL for silique dehiscence to occur later in maturation (Rajani and Sundaresan 2001; Liljegren et al. 2004; Roeder and Yanofsky 2006) (Figure 1).

It has been shown in the Triangle of U species that a portion of the mesocarp cells can undergo further lignification as the silique begins to mature (Nichol and Samuel 2024). This lignification process can be seen as early as 20 DAP to its maximum at 35 DAP (Nichol and Samuel 2024) in *Brassica* species. Within *A. thaliana* and the Brassicas, the endocarp cell layer consists of two different cell types that arise from a periclinal division giving rise to the endocarp *a* and *b* cell layers, with endocarp *b* making up small uniform lignified sclerenchyma cells and endocarp *a* located closest to the ovary space making up large rotund thin‐walled parenchymal cells (Spence et al. 1996; Rajani and Sundaresan 2001; Nichol and Samuel 2024) (Figure 1). The endocarp *a* cell layer is known to degrade within *A. thaliana* and other closely related species within the Triangle of U and the broader Brassicaceae family (Spence et al. 1996; Nichol and Samuel 2024). During this process of degradation, it is consistently observed that cells of the endocarp *a* layer closest to the replum can get lignified prior to the degradation process (Nichol and Samuel 2024) (Figure 1). The number of endocarp *a* cells that are lignified can be specific to the species, where the closest lignified endocarp *a* cell adjacent to the replum is defined as L1, with each subsequent lignified cell increasing the value numerically (Nichol and Samuel 2024). This means that the second and third endocarp *a* cells that are lignified would be defined as L2 and L3, respectively, until the maximum number of lignified cells is reached (Nichol and Samuel 2024). This number of endocarp *a* lignified cells varies from one to five depending on the stage of the maturation process and the species. In Arabidopsis, this lignification appears at 8 DAP, and by 10 DAP reaches the maximum of three cells (L1, L2, L3) lignified (Nichol and Samuel 2024) (Figure 1). Both the lignified endocarp *a* cells and the lignified mesocarp cells are thick‐walled parenchyma cells with identifiable plasmodesmatal pits (Nichol and Samuel 2024). Nichol and Samuel (2024) also proposed that the degradation of the endocarp *a* cell layer seems to follow the temporal deposition of lignification both within the endocarp *b* and *a* L1/2/3 cells within *A. thaliana* and the other *Brassica* species.

The lignification patterning within the endocarp *b* cell layer differs among other Brassicaceae members, such as *C. hirsuta* , where lignin is deposited within a V‐shaped or teardrop‐shaped pattern when looked at from a cross‐sectional point of view (Vaughn et al. 2011). Additionally, between the endocarp *a* and *b* cell layer regions, there is a large accumulation of mucilaginous pectin that is missing from *A. thaliana* (Vaughn et al. 2011). It is believed that this mucilaginous region could be thought of as an extension of the middle lamella, giving the endocarp *b* cell layer the ability to resist mechanical tensile forces (Vaughn et al. 2011). Additionally, it has been shown that in other plant species, *Litchi chinensis* and *Dimocarpus longan* , the endocarp *a* cell layer is made up of a waxy cuticular layer that is much thinner than the exocarp cell layer (Riederer et al. 2015). The endocarp *a* cell layer harbors very long‐chained aliphatic and cyclic compounds, which are believed to be the lipids associated with the makeup of this waxy layer (Riederer et al. 2015).

The difference between a silique and a silicle is the width versus the length of the fruiting body, with the silique being > 3× the length than its width and a silicle being < 3× the length than its width (Simpson 2010). Although this botanical definition for a silicle changes depending on the source that is used. The Merriam‐Webster's Dictionary definition is as follows: a silicle is a silique of nearly equal length and width (Merriam‐Webster 2025). While the Missouri Botanical Garden refers to a silicle as less than twice as long as wide (Missouri Botanical Garden 2025). Regardless of these definitions, generally, the silicle is described as a globular and/or rounded flat fruit, depending on the species; containing two loculate ovary spaces that are separated by a false partition in the form of a septum, they additionally contain two valves that converge at a replum central seam, similar to what is seen in *A. thaliana* and the other Brassicaceae (Simpson 2010; Jankowski et al. 2019). Silicle fruit morphology differs from that of the silique, where developmental transitions early on in the gynoecium give rise to the oblate spheroid shape seen in species such as *Camelina sativa* (camelina), *Thlaspi arrense* (pennycress), and *Capsella rubella* (capsella) (Eldridge et al. 2016). This development of the gynoecium continues more predominantly in *Capsella*, where unique shoulder‐like features begin to appear that create a heart‐shaped silicle (Eldridge et al. 2016). Little is known about the inner silicle morphology of species such as *Camelina*, pennycress, and *Capsella*. Recent research conducted by Chopra et al. (2020) has shown a cross‐sectional view of a pennycress silicle where the replum and valve morphology are quite similar to that of *A. thaliana* siliques with a noticeable exocarp, mesocarp, and endocarp *a* and *b* cell layer as well as lignification within the endocarp *b* cell layer and LL. The replum and valve regions are also visibly shown to come together in a DZ‐like pattern, yet whether these regions actually function similar to *A. thaliana* remains unknown, and further research is needed (Chopra et al. 2020).

## 3 Molecular Pathway Involved in Silique Dehiscence

### 3.1 Cell Differentiation Genes and Silique Development, a Nexus of Repressors That Define Layer Identity

**FIGURE 2:**
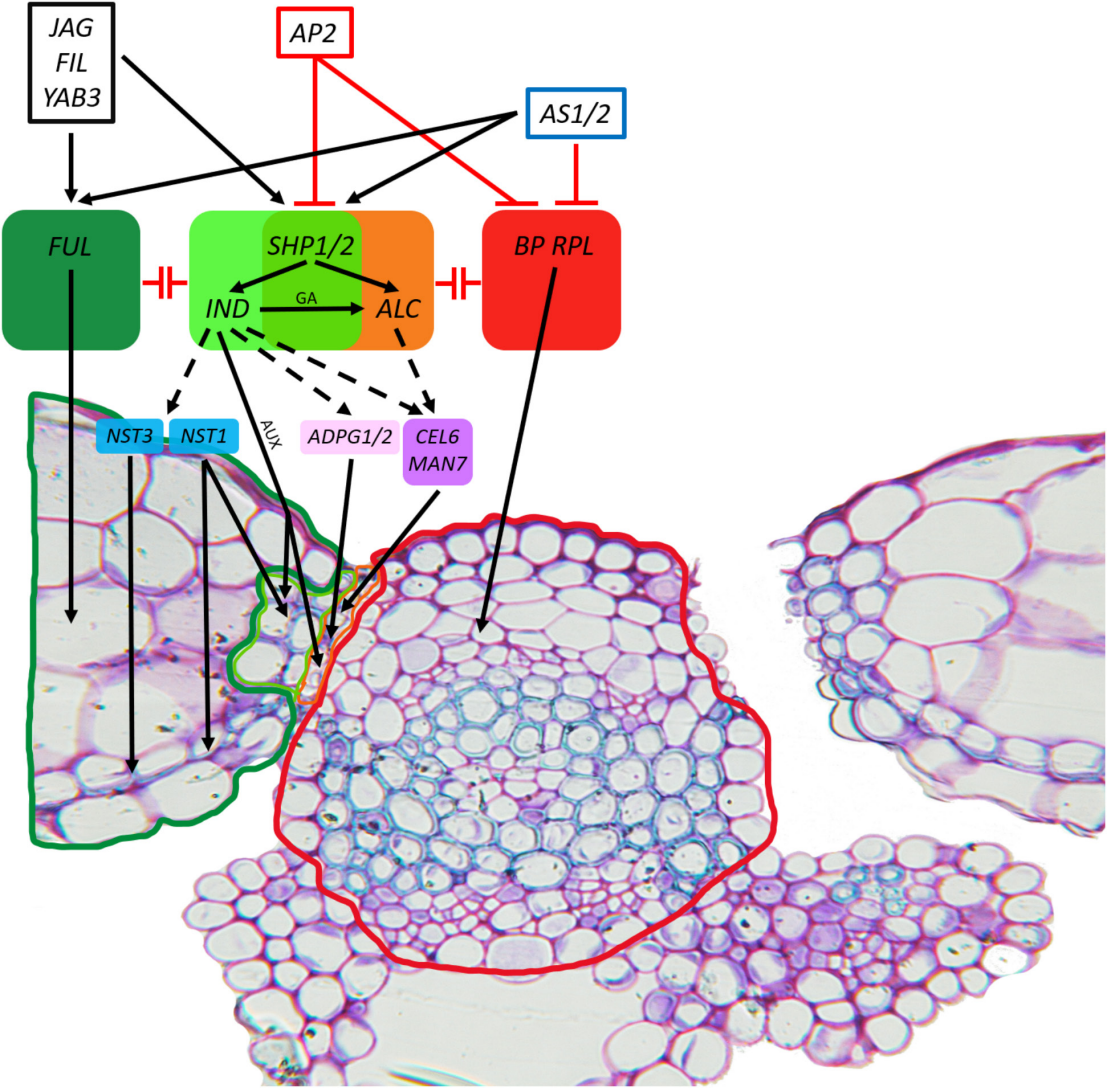
A model of *Arabidopsis* silique development and differentiation. The model depicts master regulatory transcription factors of silique development and differentiation, along with downstream interactions between opposing cell identity regulators creating expression gradients between the valve, valve margin, and replum. Known downstream cell wall modification enzymes and transcription factors are depicted. Solid lines indicate known pathways. Dotted lines indicate hypothetical interactions. The solid dark green line indicates the valve. The solid light green line indicates the lignified layer. The solid orange line indicates the separation layer. The solid red line indicates the replum. The silique is stained with toluidine blue O, with the pink/purple color representing the primary cell wall and the blue‐colored cells indicating secondary cell wall lignification.

A host of genes precisely regulate silique patterning, which has been extensively studied and been the major focus of understanding the function of fruit formation in *Arabidopsis* (Rajani and Sundaresan 2001; Liljegren et al. 2000; Ferrándiz, Gu, et al. 2000; Dinneny et al. 2005; Arnaud et al. 2010; Groszmann et al. 2011; Jaradat et al. 2014). *FRUITFULL* (*FUL*) is a member of the MCM1 AGAMOUS DEFICIENS SRF box (MADS‐box) gene family with expression throughout the plant but playing a specific role within the valve identity (Alvarez and Smyth 1997) (Figure 2). Early research into *FUL* determined that it promotes normal valve differentiation and development along with controlling the extension of the silique; this is due to the *ful‐1* knockout phenotype in *Arabidopsis* displaying shortened siliques (Gu et al. 1998; Ferrándiz, Gu, et al. 2000). The resulting *ful‐1* mutants did not create noticeable phenotypes within the replum region, displaying valve specificity because it grew to normal wild‐type size, creating a zig‐zag‐like patterned seam across the silique surface due to the shortened silique size (Gu et al. 1998). Several phenotypic changes occur within the *ful‐1* mutant, including ectopic lignification of the valve mesophyll cells and an increased number of endocarp *a* cells within the cell layer while simultaneously having reduced size and shape of the cell itself (Gu et al. 1998). The activation temporally and spatially of *FUL* within other Brassicaceae members such as *Capsella* is important in the unique heart‐shaped silicle (Eldridge et al. 2016). *Crful‐1* mutants displayed no shoulder formation and remained rounded after fertilization, indicating *CrFUL* fruit shape is modulated during the late phase when the silicle transforms from an oblate spheroid to a heart‐shaped fruit (Eldridge et al. 2016). The MADS‐box gene family members downstream of *FUL*, *SHATTERPROOF1* and *2* (*SHP1/2*) are involved in the differentiation of the DZ, promoting lignification of the LL, and promoting the formation of the valve margin (Bowman et al. 1991; Kempin et al. 1995; Purugganan 1997; Riechmann and Meyerowitz 1997; Ferrándiz, Gu, et al. 2000; Dinneny et al. 2005; Ferrándiz and Fourquin 2014) (Figure 2). It has been shown that there is no phenotypic difference between the *shp1* and *shp2* single mutants compared with wild‐type plants, while the double mutant of *shp1/2* creates indehiscent siliques (Liljegren et al. 2000). This is often attributed to the high functional redundancy of the genes, with a shared homology of 87% at the amino acid level (Ma et al. 1991; Savidge et al. 1995; Flanagan et al. 1996). In *shp1/2* double mutants, there is reduced lignification of the LL and an absence of the DZ (Liljegren et al. 2000). Studies have shown that FUL negatively regulates *SHP1* and *SHP2*, with a *35s::FUL* line showing similar phenotypic effects to that of the double mutant line of *shp1/2* with reduced lignification in the LL and a disruption in the DZ formation (Liljegren et al. 2000) (Figure 2). The overexpression line of *35s::FUL* and the double mutant of *shp1/2* both produced siliques that were indehiscent, suggesting that *FUL* and *SHP* interact antagonistically during valve margin development (Ferrándiz, Liljegren, and Yanofsky 2000) (Figure 2). Subsequent research determined that expression of *SHP1* and *SHP2* is repressed by *FUL* in the valves and by *APETALA1* (*AP1*) in the outer whorls of the flower (Ferrándiz, Liljegren, and Yanofsky 2000; Kaufmann et al. 2010).

The relationship between valve differentiation and valve margin formation is dependent on the formation of the replum. *REPLUMLESS* (*RPL*) encodes a transcription factor that belongs to the BEL1‐like family (Western and Haughn 1999). The activity of *RPL* regulates the *SHP* gene to control the development of the replum, similar to the antagonistic relationship between *FUL* and *SHP* mentioned above (Roeder et al. 2003; Ferrándiz, Liljegren, and Yanofsky 2000) (Figure 2). The *rpl* knockout had a complete loss of the replum structure, with the cells in the place of the replum resembling that of the valve margin (Roeder et al. 2003). *SHP2::GUS* lines demonstrated that the valve margin genes were likely ectopically expressed within the replum layer, converting them to valve margin cells in the *rpl* knockout (Savidge et al. 1995; Roeder et al. 2003). The triple mutant knockout with *rpl* and *shp1/2* results in the appearance of a wild‐type replum (Roeder et al. 2003). This meant that *SHP1/2* were responsible for the loss of the replum identity within the *rpl* mutant, demonstrating that the role of *RPL* is necessary to prevent the expression of *SHP1/2* within the replum region (Roeder et al. 2003) (Figure 2). A class 1 KNOX (KNOTTED‐LIKE HOMEOBOX) gene *BREVIPEDICELLUS* (*BP*), also known as *KNAT1*, is expressed within the replum, with overexpression lines having greatly enlarged replum regions, and when there is a *bp/rpl* double knockout, the resulting replum‐deficient phenotype is enhanced (Lincoln et al. 1994; Chuck et al. 1996; Douglas et al. 2002; Venglat et al. 2002; Alonso‐Cantabrana et al. 2007). The MYB transcription factor *ASYMMETRIC LEAVES1* (*AS1*), along with the LATERAL ORGAN BOUNDARY (LOB) domain protein *ASYMMETRIC LEAVES2* (*AS2*), negatively regulates *BP*, with *as1* and *as2* mutations leading to an enlarged replum region and reduced valve size (Byrne et al. 2000; Sun et al. 2002; Iwakawa et al. 2002; Shuai et al. 2002; Alonso‐Cantabrana et al. 2007; Guo et al. 2008) (Figure 2).

A downstream regulator of valve margin identity is *ALCATRAZ* (*ALC*), with *ALC* encoding a myc/basic‐helix–loop–helix (bHLH)‐related transcription factor expressed within the valve margin of the silique during silique dehiscence, associated with the development of the SL (Liljegren et al. 2000; Rajani and Sundaresan 2001; Dinneny et al. 2005; Tang et al. 2007) (Figure 2). Within *alc* mutants, the identity of the SL seems to be split between non‐lignified replum‐like cells from the midpoint of the valve to the epidermis and lignified cells from the midpoint of the valve towards the ovary space (Gu et al. 1998). The lack of an SL identity leads to the *alc* mutant having indehiscent siliques (Rajani and Sundaresan 2001). The *SPATULA* (*SPT*) gene is a conserved bHLH protein that is partially redundant to *ALC* (Groszmann et al. 2008; Groszmann et al. 2010; Groszmann et al. 2011). Because both *alc* and *spt* single knockout mutants have abnormal valve margin identity, the contributions that *ALC* and *SPT* genes have within the valve margin can be parsed through a *ful* mutant background (Liljegren et al. 2004). The *ful/alc* double mutant has a reduced ectopic valve margin despite having a functional *SPT* gene (Liljegren et al. 2004). Research done by Groszmann et al. (2011) suggests that *ALC* and *SPT* redundancy does not extend to later DZ differentiation and that *ALC* and *SPT* may have a dosage dependency within early valve margin development.

Similar to *ALC*, the highly investigated and translationally exploited *INDEHISCENT* (*IND*) gene encodes an atypical bHLH, with an alanine residue within the DNA binding region where normally a glutamic acid resides (Fisher and Goding 1992; Buck and Atchley 2003; Toledo‐Ortiz et al. 2003; Liljegren et al. 2004). *IND* is responsible for the differentiation of the valve, replum, and valve margin; layers critical for seed dispersal (Liljegren et al. 2004; Wu et al. 2006; van Gelderen et al. 2016) (Figure 2). The *ind* mutant revealed serious defects within the valve margin with no distinctive SL and no lignification within the LL, leading to an indehiscent silique phenotype (Liljegren et al. 2000; Liljegren et al. 2004). This research suggests that *IND* is important in facilitating cells to adopt a valve margin identity (Liljegren et al. 2000; Liljegren et al. 2004; Rajani and Sundaresan 2001) (Figure 2). Researchers have also discovered that the interactions between *IND* and *SPT* promote valve margin development by regulating auxin formation (Girin et al. 2011) (Figure 2). Valve margin identity genes, such as *IND*, also play a role in negatively regulating replum identity (Girin et al. 2010) (Figure 2). Additionally, *IND* promotes GA accumulation directly by affecting the gibberellin biosynthesis enzyme GA3OX1, leading to GA promoting the degradation of the DELLA protein bound to ALC, allowing ALC to act on its downstream processes, leading to SL formation (Arnaud et al. 2010; Ballester and Ferrándiz 2017) (Figure 2). A master regulator upstream of *BP*, *RPL*, *IND*, and *SHP1/2* known as *APETALA2* (*AP2*) controls the replum growth by inhibiting *BP* and *RPL* expression because in *ap2* knockouts the size of the replum increased compared with wild‐type plants (Ripoll et al. 2011) (Figure 2). Additionally, the research by Ripoll et al. (2011) determined that *AP2* negatively regulates valve margin formation through *IND* and *SHP1/2* (Figure 2). Reporter expression of *SHP* and *IND* is increased within the *ap2* knockout mutant compared with wild‐type plants, with the *ap2* mutant also leading to increases in both the size and number of lignified cells within the valve margin (Ripoll et al. 2011). The loss of replum and valve margin formation in the *35s::FUL* overexpression line was overcome by placing the overexpression line within the *ap2* background because AP2 was no longer able to suppress *BP*/*RPL* and *IND/SHP* required for replum valve margin formation, respectively (Ripoll et al. 2011) (Figure 2).

An overview of the work done above can be represented briefly here. The main tissues within the silique are the valve, valve margin, and replum (Figure 1). The expression of *FUL* is important for valve development and differentiation, additionally playing a role in the negative regulation of *SHP1* and *SHP2*, preventing valve cells from adopting valve margin cell identity (Gu et al. 1998; Ferrándiz, Liljegren, and Yanofsky 2000) (Figure 2). For proper replum development to occur, *RPL* and *BP* expression must be present to prevent the formation of valve margin cells within the replum region from *SHP1* and *SHP2* (Douglas et al. 2002; Venglat et al. 2002; Chuck et al. 1996; Lincoln et al. 1994; Alonso‐Cantabrana et al. 2007; Ripoll et al. 2011) (Figure 2). *SHP1* and *SHP2* regulate downstream genes *IND*, regulating the identity of the valve margin and subsequent differentiation into the LL and SL, and *ALC*, regulating the identity of the SL (Bowman et al. 1991; Kempin et al. 1995; Purugganan 1997; Riechmann and Meyerowitz 1997; Ferrándiz, Gu, et al. 2000; Dinneny et al. 2005; Tang et al. 2007; Ferrándiz and Fourquin 2014) (Figure 2). *FUL* negatively regulates *SHP1* and *SHP2* expression so that valve identity is not lost (Liljegren et al. 2000) (Figure 2). *FUL* and *SHP* expression is also promoted by *JAGGED* (*JAG*), *FILAMENTOUS FLOWER* (*FIL*), *YABBY3* (*YAB3*), *AS1*, and *AS2* (Sawa et al. 1999; Eshed et al. 2004; Dinneny et al. 2004; Ohno et al. 2004; Alonso‐Cantabrana et al. 2007) (Figure 2). While the expression of *BP* is negatively regulated by *AS1* and *AS2* (Alonso‐Cantabrana et al. 2007) (Figure 2). *AP2* negatively regulates *SHP* and *IND* to regulate valve margin formation (Ripoll et al. 2011) (Figure 2). Additionally, *AP2* negatively regulates *BP* and *RPL* to reduce the size of the replum (Ripoll et al. 2011) (Figure 2). A model proposed by Ripoll et al. (2011) suggests that the development of the valve, valve margin, and replum in plants is driven by the interaction of opposing factors, each promoting either valve or replum formation. These factors create gradients of activity; high valve factor activity defines valve identity, high replum factor activity defines replum identity, and valve margins form where both factors are weakly expressed. When valve factors are mis‐expressed, they repress replum and valve margin identities. Conversely, misexpression of replum factors represses valve identity.

### 3.2 Genes Associated With the Process of Silique Dehiscence

Silique dehiscence is primarily coordinated through the proper cell development and specification events from the DZ, but additional effector genes downstream add to the secondary coordinated level of silique dehiscence. Although several transcription factors define the intricate organization of the valve layer, the major process of silique dehiscence is primarily coordinated through cell wall rupture of cells in SL via cell wall degrading enzymes, which play a major role in this process. The coordinated cellular degradation of the SL is performed by degrading the cellulose, hemicellulose, pectin, and xyloglucan of the cells residing within this region. The first instance of a cell wall degrading enzyme was polygalacturonase (PG) activity documented within the DZ of *Brassica napus* by Jenkins et al. 1996 and Petersen et al. 1996. The expression patterning of PGs within the SL just prior to silique dehiscence was first demonstrated by Sander et al. 2001. In *Arabidopsis*, ARABIDOPSIS DEHISCENCE ZONE POLYGALACTURONASE1 (ADPG1) was initially suggested to be involved in silique dehiscence based on its expression pattern within the DZ (González‐Carranza et al. 2007). Additional evidence was provided by the lack of expression of *ADPG1* present within *ind* mutants (Ogawa et al. 2009). In 2009, Ogawa et al. (2009) demonstrated that two PGs, *ADPG1* and *ADPG2*, expressed within the DZ of mature siliques, were involved in silique dehiscence. The double knockout of *adpg1/2* resulted in an indehiscent silique due to the lack of pectinase activity within the DZ (Ogawa et al. 2009; He et al. 2018). Ogawa et al. (2009) also determined that the role of *ADPG1* and *ADPG2* differed in silique dehiscence due to expression levels, with *ADPG1* having a major impact compared with *ADPG2*. Additional work completed by He et al. (2018) confirmed this with an *adpg1* knockout demonstrating incomplete dehiscent siliques and an *adpg2* knockout that exhibited complete dehiscence with collapsed cells within the SL. Moreover, post‐translational modifications of PGs were demonstrated by Degan et al. (2001), where a cleavable N‐terminal domain is removed, leading to subsequent mature protein to be transported extracellularly. Pectin methylesterases may be associated with middle lamella degradation during dehiscence, although no direct evidence of this has been demonstrated (Jaradat et al. 2014).

He et al. (2018) expanded our knowledge of the various cell wall degrading enzymes functioning within the DZ of the silique with the addition of the cellulase gene *CELLULASE6* (*CEL6*) and the hemicellulase gene *MANNANASE7* (*MAN7*). The expression of *CEL6* and *MAN7* was partially dependent on the IND and ALC transcription factors (He et al. 2018). Cell wall degradation in the SL in nearly mature siliques was promoted by CEL6 and MAN7 because *cel6* and *man7* single knockouts along with the *cel6/man7* double knockout displayed an intact SL, but the siliques could still dehisce but at low rates (He et al. 2018). The degree to which the SL was degraded and the level to which the silique can dehisce was also manipulated through a set of single, double, and triple mutants of the various cell wall degrading enzymes (He et al. 2018). The effects of *CEL6*, *MAN7*, and *ADPG1* are additive because the *man7/adpg1* double knockout showed more intact cells in the SL than *adpg1* alone, while the *cel6/adpg1* double knockout had mostly intact cells at the SL (He et al. 2018). The triple mutant *cel6/man7/adpg1* closely resembled the *cel6/adpg1* double mutant (He et al. 2018). Comparatively, *ADPG1* is of critical importance in middle lamella degradation, while *CEL6* and *MAN7* are specific to cell lysis, with *CEL6* having a greater role in this process than *MAN7* (He et al. 2018). RNAi lines of *MAN7* in *B. napus* were also shown to promote silique indehiscence (Li et al. 2021). Xyloglucan endotransglycosylase was determined to be another cell wall degrading enzyme shown within the DZ of *B. napus* siliques (Fry et al. 1992; Roberts et al. 2000). The antagonistic relationship between known plant hormones, ethylene and auxin, aids in promoting and inhibiting the DZ through cell separation timing, respectively (González‐Carranza et al. 1998; Child et al. 1998). A decrease in the auxin concentration increases cellulase activity within the DZ, while the application of exogenous auxin analogs inhibits cellulase activity and pectinase secretion, ultimately delaying DZ cell separation (Degan et al. 2001).

Another essential process necessary to allow for proper seed dispersal occurs through *NAC SECONDARY WALL THICKENING PROMOTING FACTOR 1 and 3* (*NST1/3*) genes, which have been shown to play important roles in the SCW thickening within specific tissues (Mitsuda et al. 2005; Mitsuda et al. 2007; Mitsuda and Ohme‐Takagi 2008). NST1 promotes SCW thickening and formation within the LL of the valve margin and the endocarp *b* cell layer within *A. thaliana* (Mitsuda and Ohme‐Takagi 2008). Research done by Zhong et al. (2006) described a role for the *SECONDARY WALL–ASSOCIATED NAC DOMAIN PROTEIN‐1* (*SND1*) in endocarp *b* lignification. It should be noted that *NST3* and *SND1* are the same gene but were designated differently by the two groups (Mitsuda and Ohme‐Takagi 2008). SND1/NST3 promotes SCW thickening and formation within the endocarp *b* cell layer (Mitsuda and Ohme‐Takagi 2008; Zhong et al. 2006). The *NST1* and *SND1/NST3* genes function as the first layer of the regulatory network for SCW deposition, classified by Zhang et al. (2018). The double knockout of *nst1/3* leads to a silique with a complete lack of lignification within the LL and endocarp *b* cell layer, leading to silique indehiscence (Mitsuda and Ohme‐Takagi 2008). Studies conducted by Zhong et al. (2007) and McCarthy et al. (2009) revealed that *MYB46* and *MYB83* are direct targets of SND1/NST3, which play a key role in activating SCW biosynthesis. This was demonstrated through the knockout lines of *myb46* and *myb83*, where a marked reduction in SCW size was observed (Zhong et al. 2007; McCarthy et al. 2009).

In *C. hirsuta* , unique asymmetrical polar deposition of lignin within the endocarp *b* cell layer is controlled through *LACCASES* (*LACs*) *4*, *11*, and *17* (Pérez‐Antón et al. 2022). LAC4/11/17 localize in the zones with asymmetric lignification within endocarp *b* cells (Pérez‐Antón et al. 2022). Interestingly, when LAC4 and LAC17 were expressed under their native promoters in *A. thaliana* , non‐polar localization of lignification was seen (Pérez‐Antón et al. 2022). A known transcription factor, *SQUAMOSA PROMOTER‐BINDING PROTEIN‐LIKE 7* (*SPL7*), that regulates copper (Cu) homeostasis, when mutated, was shown to limit the range of the explosive seed dispersal force (Pérez‐Antón et al. 2022). This was due to *spl7* exhibiting a silique bulking phenotype that affects the amount of stored potential elastic energy for explosive seed dispersal (Pérez‐Antón et al. 2022). This Cu‐dependency through LAC4 and LAC17 determines the endocarp *b* lignification within *spl7* mutants. Cu homeostasis through SPL7 is required to activate Cu‐requiring laccases to regulate lignification within the endocarp *b* cell layer, leading to the explosive seed dispersal, demonstrating mineral nutrition with polar lignification in endocarp *b* (Pérez‐Antón et al. 2022).

## 4 Understanding the Process of Silique Dehiscence

**FIGURE 3:**
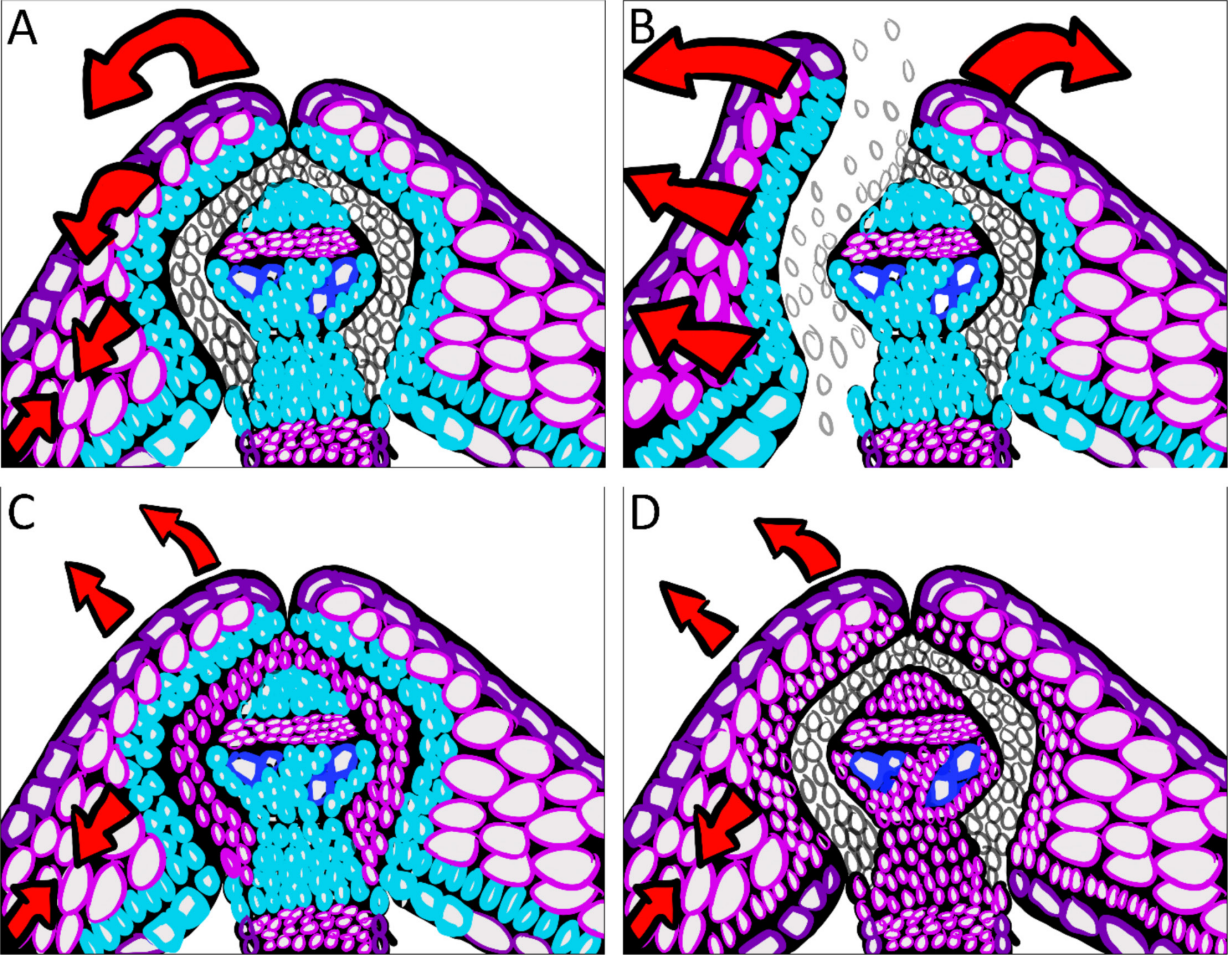
An illustrative figure of a wild‐type *Brassica napus* silique DZ and mutant variations. The siliques are false‐colored to mimic toluidine blue O staining, with the pink/purple color representing the primary cell wall and the blue‐colored cells indicating secondary cell wall lignification (A–D). The large red arrows represent forces applied onto the silique structure (A–D). A representative wild‐type *Brassica napus* silique that has not undergone dehiscence (A). A representative *Brassica napus* wild‐type silique that has undergone dehiscence within the DZ, illustrates the separation of the valve from the replum (B). A mutant *Brassica napus* silique that no longer has proper cell wall and middle lamella degradation due to hypothetical knockouts in cell wall degrading enzymes (C). A mutant *Brassica napus* silique that no longer has secondary cell wall deposition within the endocarp *b* cells and lignified layer due to hypothetical knockouts in lignification deposition/patterning (D).

The process of silique dehiscence is essential for the proper dispersal of seeds at the end of a dehiscent plant's lifecycle (Howe and Smallwood 1982; Howe and Miriti 2004; Moles and Westoby 2006). Silique dehiscence occurs through the spatial and temporal relationships of many silique morphology genes and downstream cell wall degrading enzymes as mentioned above (Rajani and Sundaresan 2001; Liljegren et al. 2000; Ferrándiz, Gu, et al. 2000; Dinneny et al. 2005; Arnaud et al. 2010; Groszmann et al. 2011; Jaradat et al. 2014) (Figure 2). Once the identity of the replum region and valve region has been established, the silique of *Brassica* species and *A. thaliana* will undergo what we classify here as two priming events that occur almost simultaneously to allow for dehiscence to occur (Squires et al. 2003) (Figure 3a). The first priming event is the development of the DZ with lignification occurring within the LL and the cell wall degrading enzymes acting on the SL (Ferrándiz et al. 1999; Liljegren et al. 2004; Roeder and Yanofsky 2006) (Figure 3a). While lignification of the LL allows for additional stability of the silique, it also allows for tensile forces from upper drying layers to converge on the LL (Ferrándiz et al. 1999; Liljegren et al. 2004; Roeder and Yanofsky 2006) (Figure 3a). Cellular degradation of SL weakens this particular section of the silique, allowing for a location where the splitting of the valve away from the replum can occur (Ferrándiz et al. 1999; Liljegren et al. 2004; Roeder and Yanofsky 2006) (Figure 3a,b). These two processes in conjunction act as the primary priming step of silique dehiscence. The second priming step occurs through the lignification of the endocarp *b* and endocarp *a* L1–3 cells and the mesocarp layer's additional lignification (Spence et al. 1996; Rajani and Sundaresan 2001; Nichol and Samuel 2024) (Figure 3a). Once these two priming steps are complete, the silique is ready to begin the process of dehiscence (Figure 3b). Near the end of the silique lifecycle, depicted at Stage 18 using the classical staging classification or after 12 DAP/DPA using the alternate staging classification, the silique begins the process of drying (Spence et al. 1996; Rajani and Sundaresan 2001; Nichol and Samuel 2024). Through the process of drying, the collapsing, non‐lignified cells of the mesocarp layer begin to create tensile forces that apply to the radial walls of the cell because the tangential walls start to collapse (Vaughn et al. 2011). The tensile forces created from the mesocarp layer are represented by the smaller two arrows facing each other in Figure 3a–d. The tensile force is amplified because of the LL, the endocarp *a* L1–3 cells, and the rigidity of the endocarp *b* cell layer (Vaughn et al. 2011; Nichol and Samuel 2024). This is visualized in Figure 3a. where the large red arrow on top of the replum is the active mechanical force being applied to the valve. The mechanical tensile forces pull the top of the valve towards the middle of the valve region in a spring‐like fashion (Figure 3a). Once the applied mechanical force is large enough, the weakest point of the silique, which is the SL, will begin to separate because there is no longer a strengthened cell wall or middle lamella providing support (Ferrándiz et al. 1999; Liljegren et al. 2004; Roeder and Yanofsky 2006) (Figure 3b). Once the SL separates, the silique will begin to dehisce, and subsequent seed dispersal can occur (Ferrándiz et al. 1999; Liljegren et al. 2004; Roeder and Yanofsky 2006) (Figure 3b).

There are commonalities and differences in silique dehiscence observed in other Brassicaceae members, wherein *C. hirsuta* , priming events occur similar to *Brassicas* and *A. thaliana* , as mentioned above, with lignification of the endocarp *b* cell layer and LL along with degradation of the secondary cell wall within the SL as demonstrated by Cullen and Hay (2024). *C. hirsuta* differs in having asymmetric polar deposition of lignification in the form of a V‐shaped or teardrop‐shaped endocarp *b* and a mucilaginous layer deposited within the endocarp *a* and *b* cell layer (Vaughn et al. 2011; Cullen and Hay 2024). *C. hirsuta* undergoes similar outer pericarp drying with the non‐lignified cells of the mesocarp layer creating similar tensile forces across the radial walls (Vaughn et al. 2011). Due to the V‐shaped lignification of the endocarp *b* cell layer, collapse of the upper endocarp *b* cell wall leads to an ultrafast coiling of the silique valve, releasing the seeds in an explosive manner (Cullen and Hay 2024). In camelina and pennycress, although little is known about the mechanisms behind silicle dehiscence, we can infer from the similarities to *A. thaliana* in cell layer morphology and patterning, that the overall mechanism functions in a similar manner. However, ultimately, more research in this area is needed to verify the developmental patterning and inner silicle morphology to further determine the actual mechanisms at play.

The absence of either of the priming effects disrupts the action of silique dehiscence. This is either through disruptions of the cell wall degrading enzymes or the lignification pathway and downstream genes associated with silique lignification (Ogawa et al. 2009; Mitsuda and Ohme‐Takagi 2008; He et al. 2018) (Figure 3c,d). If the cell wall degrading enzymes mentioned previously in Section 3.2 are disrupted as illustrated in Figure 3c, the SL no longer contains weakened cells because the cell wall and middle lamella are left intact (Ogawa et al. 2009; He et al. 2018). This creates a scenario where the mechanical tensile forces from mesocarp drying are insufficient to break open the SL and split the valve from the replum, leading to a partial or fully indehiscent silique (Ogawa et al. 2009; He et al. 2018) (Figure 3c). If the lignification pathway or downstream genes associated with silique lignification deposition are disrupted, as illustrated in Figure 3d, the mechanical tensile forces are insufficient to break open the SL, even though the SL has undergone proper cell wall degradation (Mitsuda and Ohme‐Takagi 2008). The lignification of the LL, endocarp *a* L1–3 cells, and endocarp *b* cells are necessary because the provided rigidity of the lignified cells is essential for mechanical tensile forces to be applied to the valve (Mitsuda and Ohme‐Takagi 2008; Nichol and Samuel 2024) (Figure 3d). This is because the applied force on the radial wall is dispersed throughout the valve and not focused on the LLs (Mitsuda and Ohme‐Takagi 2008) (Figure 3d). This creates a scenario where the valve cannot separate from the replum, leading to a partial or fully indehiscent silique (Mitsuda and Ohme‐Takagi 2008) (Figure 3d). Any approach to creating shatter tolerance should consider establishing the necessary valve identities so that the siliques are shatter tolerant and not shatter resistant. In simple terms, creating a silique that is shatter resistant from loss of DZ identity will be difficult to break open by the harvesting machinery due to the lack of the SL, leading to separation in planes other than the valve margins to release the seeds. This approach, although can result in shatter‐resistant siliques, may not result in the yield advantage as the valve is unable to open along the natural plane to release all the seeds. The better approach would be to create strong, indehiscent siliques with proper valve identities that can be broken open with added pressures from the harvesting machinery.

## 5 Effects of Shattering on Oilseed Crops

Weather and climate‐related abiotic factors such as periods of drought and hail can exacerbate the natural dehiscence process, resulting in lost yield (Bara et al. 2013; Steponavičius et al. 2019). A previous study by Kutcher et al. (2010) has reported that hot and dry climates can reduce the number of fertile siliques on the main branch, seed weight, and number of seeds per silique. Additionally, these conditions cause tensile force to build up rapidly, resulting in silique shatter. Similar oilseed crop harvest loss percentages are seen throughout Canada, Europe, and China, with average losses due to shatter hovering around 6%–10% of the total yield, with this reaching as high as 20% (Kadkol et al. 1984; Price et al. 1996; Pekrun et al. 1998; Gulden et al. 2003; Weber et al. 2009; Pari et al. 2012; Peltonen‐Sainio et al. 2014; Cavalieri et al. 2016; Kuai et al. 2016). In 2021, the Canadian prairies experienced the greatest reduction in yield due to intense drought conditions (StatisticsCanada 2024). Canola yields decreased by 45%, 31%, and 28% in Saskatchewan, Alberta, and Manitoba, respectively (StatisticsCanada 2024). Specifically, in 1996, up to 50% of canola yield was lost due to what is agriculturally known as “pod shatter,” from a combination of non‐ideal weather conditions, natural dehiscence, and mechanical harvesting mechanisms (Price et al. 1996; Maity et al. 2021). By 2003, 6% of canola yield was lost, which amounted to roughly 3000m^2^ of seeds, all of which would then become volunteer plants and would have to be treated as pests (Gulden et al. 2003; Maity et al. 2021). The reduction in yield loss has been attributed to the development of shatter‐tolerant varieties through a combination of traditional breeding, hybridization, and other genetic modification (GM) techniques, although even a 6% loss of yield approximately results in a $1.8B CAD loss in revenue in Canada (The Canola Council of Canada 2024; Alberta Canola 2021; CBAN 2024).

*Camelina* has been receiving much attention in the past decade as it has shown potential for becoming a valuable oilseed crop that is not susceptible to common Brassica pests with a desirable seed oil composition, making it applicable for both feed and non‐feed uses (i.e., livestock feed and biodiesel) (Guy et al. 2014; Zanetti et al. 2021). Despite these economically valuable traits, it remains a species susceptible to drought stress, with yield losses increasing to 29% when combined directly due to silicle dehiscence from mechanical disturbance produced by the combine (Sintim et al. 2016). Similarly, pennycress yield loss significantly increases to 70% during harvest due to low moisture content within the silicle at maturity (Cubins et al. 2019). This translates to a loss of 300 times the seeding rate of pennycress, and because each pennycress crop can lose up to 15,000 seeds from shatter, it results in large‐scale growth of volunteer plants for farmers to then manage (Cubins et al. 2019).

## 6 Mitigating Silique Dehiscence

### 6.1 Classical Methods for Reducing Silique Dehiscence

Traditionally, the starting point at which crop growers will attempt to minimize seed loss through silique dehiscence during harvest is by optimizing the time of harvest and the speed of the combine (Price et al. 1996; Jeschke 2017; The Canola Council of Canada 2024). Checking the seed color and moisture content of siliques is also necessary to learn of silique maturity and prevent delayed harvest. Post‐maturity harvesting makes oilseed crops significantly susceptible to shattering from any mechanical stress caused by wind, rain, and combine use (Sintim et al. 2016). Specifically, it is recommended to harvest pennycress when it reaches physiological maturity about 2 weeks prior to harvest when silicle moisture is low enough for an efficient harvest, and this could mitigate large yield losses to shatter mentioned earlier (Cubins et al. 2019). In *Brassica* cultivation, fruit sealants consisting of various latex and resin ingredients have been used to prevent siliques from shattering by limiting water movement (Serafin‐Andrzejewska et al. 2021). This allows seeds to further develop and mature in an even manner (Serafin‐Andrzejewska et al. 2021). Additionally, swathes are placed beneath the plants for 7–14 days prior to combining, instead of direct cutting or combining (Price et al. 1996; Summers et al. 2003; Ogutcen et al. 2018). This has demonstrated increased results in minimizing seed loss (Price et al. 1996; Summers et al. 2003; Ogutcen et al. 2018). This method also prevents issues of uneven seed maturity and harvesting of low‐quality seeds (Ogutcen et al. 2018). Desiccant sprays are also routinely applied after swathing to increase the rate of maturation (Price et al. 1996; Summers et al. 2003).

### 6.2 Reducing Silique Dehiscence: Lignification Approaches

One promising approach to mitigate the seed dispersal mechanisms problem is by increasing lignification, which enhances the structural integrity of silique walls, making them less prone to shattering. Chu et al. (2022) determined that a *B. napus* single recessive gene controlled a lignified bridge between the two LLs of the silique, leading to indehiscence and high shatter tolerance. Conversely, when lignification is completely absent within the silique walls, such as in the *nst1/3* double knockout mutant, the silique no longer has mechanical tensile forces being applied, leading to indehiscent siliques (Mitsuda and Ohme‐Takagi 2008). This may also be the case for the lignin biosynthetic transcription factors of *MYB46* and *MYB83*, where a potential double knockout mutant could lead to silique indehiscence due to a complete loss of lignification within the silique walls. Focusing efforts on the genetic manipulation of the lignification pathway and lignin biosynthesis genes can allow for enhancing the mechanical strength of fruiting bodies. Hypothetically, increasing lignification in mesocarp could lead to a reduction in the tensile forces from the mesocarp drying, leading to reduced shatter. Conversely, eliminating lignification in the endocarp *b* can also lead to indehiscent siliques by distributing these mechanical tensile forces or preventing the tension from the forces from impinging on the LL to break the silique. This structural reinforcement, whether through increased lignification or complete elimination, can help ensure siliques remain intact under various environmental conditions. Ultimately, this approach minimizes seed loss and improves overall crop yield and stability.

### 6.3 Reducing Silique Dehiscence: Non‐Lignification Approaches

Similar to lignification, bioengineering plants using non‐lignifying approaches is another potential strategy to mitigate seed dispersal mechanisms. Research within this area has focused primarily on the manipulation of the main genes associated with morphological development and cell identity. Knockouts within the *IND*, *SHP1/2*, and *ALC* have been shown to lead to indehiscent siliques (Ferrándiz, Liljegren, and Yanofsky 2000; Liljegren et al. 2000; Rajani and Sundaresan 2001; Liljegren et al. 2004). While overexpression of *FUL* also leads to an indehiscent phenotype (Ferrándiz, Liljegren, and Yanofsky 2000). This work has led to the development of agricultural technologies that resulted in both accepted patents and patents pending approval. In 2008, an original patent (US20110030106A1) was filed by Bayer CropScience NV. This patent was based on a full phenotypic knockout *IND*. This technology was later patented under US9475849B2, with the current assignee being BASF Agricultural Solutions Seed US LLC, with this additional patent extending the full phenotypic knockout of *IND* to two genes within *B. napus* plants. In regards to pennycress, two recent patent filings within 2023, (US20240065193A1) and (US20240147929A1), have been filed by the University of Minnesota and Covercress Inc., respectively, with both patents having a pending status for the implementation of *IND* knockouts. Patent (US20230057587A1) has been filed with a pending status for gene knockouts within *SHP* by Cibus Europe BV, Cibus US LLC. This claim has been extended to many cash crop varieties, such as *B. napus* , *Brassica rapa* , *Brassica oleracea* , *Brassica juncea* , *Brassica* species, *Raphanus sativus* , *Pisum sativum* , *Phaseolus vulgaris* , *Lens culinaris* , *Glycine max* , and Fabaceae species. Limagrain Europe SA has filed pending patents in both Canada and the United States (US20210169029A1 and CA2994405A1; WO2017025420A1) trying to create a shatter‐tolerant line (POSH+) through manipulation of a single nucleotide polymorphism within the *FUL* gene of *B. napus* , *B. juncea*, *and B. rapa* through the addition of a *Raphanus* genomic fragment. These examples are a small subset of patent filings that demonstrate agricultural applications of shatter‐tolerant crop varieties based on the genes associated with development and cell identity.

There is potential for future efforts focusing on creating agricultural resilient crops conferring shatter tolerance through the disruption of cell wall degrading enzymes within the DZ. Research done by Li et al. (2021) has provided insights using RNAi technology targeting *MAN7* within *B. napus* creating lines that are indehiscent. Future genetic approaches could use *CEL6* and *ADPG1/2* as potential targets for silique shatter–tolerant crops because it is known within *Arabidopsis* that knockouts within these genes lead to indehiscent siliques (Ogawa et al. 2009; He et al. 2018).

## 7 (Conclusion) Future Applications for Food Security and Sustainability

Silique dehiscence in *Arabidopsis* involves a tightly coordinated set of events regulated by master controllers of morphological development and cell identity, followed by secondary cell wall‐activating transcription factors and cell wall‐degrading enzymes. Each level of regulation is crucial for natural seed dispersal. However, from an agricultural and economic perspective, silique dehiscence is unfavorable as it leads to significant pre‐harvest seed loss, reducing crop yield and profitability in crops like canola, pennycress, and *Camelina*.

Research into seed dispersal mechanisms has enabled the development of more efficient and resilient crop varieties, addressing critical challenges in oilseed crop loss. Researchers have successfully created genetically modified plants within Brassicaceae species that exhibit indehiscent siliques by reducing allele function within genes such as *IND*, *ALC*, *FUL*, and *SHP*, with patents pending approval. Future genetic approaches may focus on increasing silique strength by enhancing secondary cell walls through increased lignification or reducing cell wall‐degrading enzymes to achieve shatter tolerance in oilseed crops.

Although the current knowledge presented is limited to Brassicaceae, it can be extended to other crop varieties depending on their own similarities of silique/silicle morphology to the Brassicaceae family. Overall, the understanding of silique dehiscence has laid the groundwork for broader applications across diverse oilseed crops and beyond.

## Author Contributions

J.N. and M.S. conceived the idea. J.N. wrote a majority of the work. S.D. wrote Sections 5 and 6.1. J.N., S.D., and M.S. all contributed to editing.

## Conflicts of Interest

The authors declare no conflicts of interest.

## Data Availability

No restrictions on the data.
